# Longitudinal Clinical, Histopathologic, and Immunologic Findings in Cats with Feline Chronic Gingivostomatitis Following Partial-Mouth Extractions: A Pilot Prospective Study

**DOI:** 10.3390/vetsci13101038

**Published:** 2026-10-01

**Authors:** Agustina Algorta, Laura Tejera, Belén Varela, Gabriela Turini, Micaela Medina, Juan Eguren, María Noel Glausiuss, Ana Whyte, José Manuel Verdes, Kevin Yaneselli

**Affiliations:** 1 Unidad de Inmunología e Inmunoterapia, Facultad de Veterinaria, Universidad de la República, Montevideo 13000, Uruguay; agustinaalgorta@gmail.com (A.A.); micaelamedinag@gmail.com (M.M.); 2 Servicio de Odontoestomatología, Unidad de Clínica de Pequeños Animales, Facultad de Veterinaria, Universidad de la República, Montevideo 13000, Uruguay; gaturini@gmail.com (G.T.); juanmeguren@gmail.com (J.E.); marianoelglausiuss@gmail.com (M.N.G.); 3 Unidad de Imagenología y Laboratorio de Análisis Clínico y Endocrinología y Metabolismo Animal, Facultad de Veterinaria, Universidad de la República, Montevideo 13000, Uruguay; dra.lauratejera@gmail.com; 4 Unidad de Patología, Facultad de Veterinaria, Universidad de la República, Montevideo 13000, Uruguay; belenvarela42@gmail.com (B.V.); jose.verdes@fvet.edu.uy (J.M.V.); 5 Unidad de Cirugía y Anestesia de Pequeños Animales, Facultad de Veterinaria, Universidad de la República, Montevideo 13000, Uruguay; 6 Departamento de Patología Animal, Facultad de Ciencias Veterinarias, Universidad de Zaragoza, 50013 Zaragoza, Spain; awhyte@unizar.es

**Keywords:** gingivostomatitis, inflammation, dental extraction, histology, cat

## Abstract

Feline chronic gingivostomatitis (FCGS) is a painful inflammatory disease of the oral cavity in which partial-mouth or full-mouth extraction (PME or FME) is considered the standard treatment. However, longitudinal information regarding histopathologic and local immunologic changes after surgery remains limited. The aim of this pilot prospective study was to assess the clinical, hematologic, biochemical, histopathologic, and immunologic evolution of cats with FCGS following PME. Five cats diagnosed with FCGS were enrolled and followed longitudinally. Clinical response was assessed in the five cats using the Stomatitis Disease Activity Index (SDAI), together with hematologic, biochemical, and retroviral testing. Biopsies of the caudal oral mucosa were collected from three cats before surgery and 30 days after surgery for histopathologic evaluation using a previously described inflammatory scoring system. Immunolabeling of CD20, FoxP3, and IL-17 was quantified by digital image analysis. Clinical scores improved after PME, although the magnitude of the response varied among individuals. Persistent non-regenerative anemia and marked monocytosis were observed in cats positive for feline leukemia virus and/or feline immunodeficiency virus. Hyperglobulinemia was the most common biochemical abnormality. Histopathologic examination revealed moderate-to-marked lymphoplasmacytic inflammation associated with epithelial hyperplasia, edema, and, in the most severe cases, spongiosis and necrosis. Sequential biopsies demonstrated reductions in histologic inflammatory scores in two of three cats. CD20 immunolabeling decreased post-PME, whereas FoxP3 and IL-17 expression showed marked interindividual variability. This pilot prospective study provides preliminary observations on tissue changes in the early stages following PME.

## Simple Summary

Feline chronic gingivostomatitis (FCGS) is a severe inflammatory disease of the oral cavity that causes pain, difficulty eating, and reduced quality of life. Dental extractions are considered the standard treatment, but little is known about how the oral tissues and local immune response change after surgery. This pilot prospective study followed five cats after treatment, evaluating their clinical and blood parameters, with three cats also being evaluated for histopathologic changes and local immune cell markers. Most cats showed clinical improvement after dental extractions. Reduced inflammation was observed in oral biopsies. Expression of B-cell markers in the affected tissues was reduced after dental extraction. However, changes in other immune markers varied among individuals. These findings provide preliminary information on the local tissue response following standard surgical treatment and improve our understanding of this chronic inflammatory disease.

## 1. Introduction

Feline chronic gingivostomatitis (FCGS) is a painful, debilitating, and multifactorial inflammatory disease characterized by severe inflammation affecting the oral mucosa that markedly decreases the quality of life of affected cats [1,2,3,4,5]. Lesions may involve the gingival, alveolar, and buccolabial mucosae, and most characteristically, the caudal oral mucosa lateral to the palatoglossal folds [3,4,5]. Extraoral manifestations, including esophagitis and tracheobronchial inflammation, have also been described, further supporting the concept that FCGS is associated with widespread mucosal inflammation [6,7]. Histologically, the disease is characterized by a dense lymphoplasmacytic infiltrate accompanied by variable numbers of neutrophils and macrophages. Immunophenotypic studies have demonstrated the presence of multiple immune cell populations within affected tissues, including CD20+ B lymphocytes, CD3+ T cells (including CD4+ helper and CD8+ cytotoxic subsets), and FoxP3+ regulatory T cells [8,9]. More recently, transcriptomic and immunological studies have suggested that FCGS is characterized by persistent immune dysregulation involving alterations in T-cell function and inflammatory pathways, supporting the hypothesis that the disease represents an aberrant host response [2,8,10].

Surgical treatment through partial-mouth extraction (PME) or full-mouth extraction (FME) remains the current standard of care, although approximately 30% of cats exhibit partial clinical responses or remain refractory to treatment [1,11]. Previous investigations evaluating treatment efficacy have primarily focused on clinical outcomes [11,12,13,14], whereas more recent studies have explored alterations in the oral microbiome, systemic inflammatory biomarkers, and transcriptomic profiles associated with disease progression and treatment response [15,16,17,18,19]. In particular, transcriptomic analysis of circulating CD8+ T cells demonstrated an exhaustion-like phenotype and impaired mitochondrial bioenergetics, while cats evaluated after FME and mesenchymal stem cell (MSC) therapy showed partial recovery of these molecular signatures [19].

Despite these advances, relatively little is known regarding the evolution of the inflammatory infiltrate and local immune response within the oral mucosa following FME or PME. Most studies characterizing the oral immune microenvironment have focused either on disease pathogenesis or evolution in cats refractory to FME undergoing MSC therapy [2,8,20,21,22]. Although reductions in lymphoplasmacytic infiltration have been reported in treatment responders receiving cell-based therapies, longitudinal information describing the histopathologic and immunological changes after standard surgical treatment remains limited. Owing to the practical difficulties associated with obtaining sequential oral biopsies, the local tissue response to dental extractions has been insufficiently characterized.

The aim of the present prospective study was to describe the clinical, hematologic, biochemical, histopathologic, and local immunological changes that occur after PME in cats with FCGS.

## 2. Materials and Methods

### 2.1. Study Design

This pilot prospective longitudinal study was conducted to characterize the clinical, hematologic, biochemical, histopathologic, and local immunological changes that occur after PME in cats diagnosed with FCGS (Figure 1). The inclusion criteria were as follows: cats presenting inflammatory lesions compatible with FCGS, without oncologic or immune-mediated disease. Selected patients exhibited caudal oral mucositis and alveolar mucositis involving the premolar and/or molar regions, with no involvement of the canine or incisor regions. There were no restrictions regarding breed, age, body weight, or feline immunodeficiency virus (FIV) or feline leukemia virus (FeLV) status. Written informed consent was obtained from all owners before enrollment. Corticosteroid therapy, when present, was discontinued at least 20 days prior to surgery and was not permitted during the study period. No cat received a long-acting injectable glucocorticoid. NSAIDs, pregabalin, gabapentin, and opioids were allowed during the study period. Cats meeting the inclusion criteria underwent PME, comprising extraction of all the premolar and molar teeth. Before surgery, cats received antibiotics (amoxicillin–clavulanic acid (15 mg/kg BID) or spiramycin–metronidazole (75,000 IU/kg and 12.5 mg/kg, respectively)) and NSAIDs (meloxicam (0.1–0.02 mg/kg SID)). Antibiotics were maintained from 7 to 10 days after surgery. Intraoral radiographs were obtained before PME, but post-extraction dental radiographs were not routinely obtained; they were only performed when root fracture occurred to confirm complete root removal. Between 7 and 15 days after surgery, the cats received amoxicillin–clavulanic acid and meloxicam (dose reduced from 0.1 mg/kg to 0.03 mg/kg SID), as well as pregabalin (2 mg/kg SID or BID). The cats were evaluated on the day of PME (D0); at 7 (D7), 15 (D15), 30 (D30), and 60 (D60) days; and monthly thereafter until 6 months post-treatment (D180). Evaluations were performed without sedation at all time points, except at D0 and D30, when three cats were anesthetized for biopsy sampling.

### 2.2. Clinical Evaluation

Clinical disease severity was evaluated using the Stomatitis Disease Activity Index (SDAI) scoring system, considering both investigator and owner evaluations, and ranged from 0 (healthy) to 30 (severe) [12,23]. Scoring was performed at every follow-up. Investigators scored the lesions (0–3, from No Lesion to Severe), and the cats’ owners scored the cat’s appetite, activity level, grooming behavior and perceived oral comfort. The percentage of SDAI improvement was calculated using the following formula: [(initial SDAI D0 − post-treatment follow-up SDAI)/initial SDAI D0] ×100; the threshold for improvement was >12% following previous studies [23]. A reduction of >50% compared to the initial SDAI score was considered good improvement, >30–50% was considered moderate improvement, 12–30% was considered mild improvement, −12 to <12% was considered no improvement, and less than −12% was considered worsening [23].

### 2.3. Blood Parameters

Complete blood counts (ProCyte One analyzer, Idexx, Maine, USA) and serum biochemical (CM320, Weiner lab, Buenos Aires, Argentina) analyses were performed at predetermined time points throughout follow-up. Patients were considered anemic when at least two of the following erythrocyte parameters were below the established reference ranges for the species: total red blood cell count, hematocrit, and/or hemoglobin concentration. Anemia was classified as regenerative or non-regenerative according to the absolute reticulocyte count [24]. Retroviral infection status was determined using commercially available point-of-care assays for FeLV antigen and FIV antibodies (FASTest FIV/FeLV, Megacor Diagnostik GmbH, Hörbranz, Germany).

### 2.4. Evaluation of Local Inflammation

A 0.5 cm full-thickness biopsy was collected under general anesthesia from the caudal oral mucosa lateral to the palatoglossal folds of three cats at D0 and D30. The biopsy sample was collected from one side of the oral cavity at D0 and from the contralateral side at D30. Histopathologic and immunohistochemical (IHC) analyses were performed on formalin-fixed, paraffin-embedded specimens. Tissue samples were fixed in 4% buffered formaldehyde, processed, and sectioned into 4 μm thick sections. To assess inflammation, the sections were stained with hematoxylin and eosin (H&E). The degree of inflammation was evaluated using a scoring system for the severity of the microscopic inflammatory changes (Grades 0–3, from normal to severe) [9]. Histological images were visualized and acquired using a transmitted light microscope (Olympus BX41, Olympus Corporation, Tokyo, Japon) equipped with a high-resolution digital camera and capture software (AmScope MU853B USB 3.0 high-speed digital camera, CA, USA). The sections were also scanned using a whole-slide scanner (Motic EasyScan One scanner, Motic China Group Co., Ltd., Xiamen, China) at a focal plane with default settings and a magnification of 400× (image resolution: 0.25 µm/pixel) and visualized using a viewer (Motic ‘DSAssistant’, Motic VM V1 Viewer 2.0, version 2019-08-02, China) and scanning software (Motic ‘EasyScanner’, Hong Kong, China). Histopathologic evaluation was performed by four observers blinded to the patient and sampling time point, with final scores assigned by consensus.

To assess the type of cellular infiltrate, expression of CD20, FoxP3, and IL-17 antigens was evaluated. For this, tissue sections were deparaffinized with xylene and rehydrated through a graded ethanol series. A 3% hydrogen peroxide blocking solution was applied, followed by heat-induced antigen retrieval in a steamer, with slides submerged in 0.01 M citrate buffer (pH 6) in a polypropylene Coplin jar (Deltalab, Barcelona, Spain) for 30 min. IHC conditions were optimized using feline lymph nodes and caudal oral mucosa as positive controls. Negative controls were performed by replacing the primary antibody with phosphate-buffered saline (PBS). Isotype controls were not performed. Manufacturer recommendations were followed with minor modifications. The primary antibodies used in this study are listed in Table 1. The selected CD20 antibody [25] and FoxP3 clone (221D) [26] have been used in feline tissues. IHC detection of IL-17A has previously been reported in other feline tissues using a different antibody [27]; however, this does not constitute validation of the antibody used in the present study for feline tissue. Each primary antibody was applied to the tissue, which was then incubated overnight at 4 °C in a humid chamber. The detection system (Mach 4 Universal HRP-Polymer Detection System, Biocare Medical, CA, USA) and streptavidin–peroxidase were applied. Labeling was visualized using DAB solution for 3–5 min, and then the sections were counterstained with Mayer’s hematoxylin, dehydrated, and mounted. Histological images were acquired as previously described.

Immunolabeled areas were quantified using digital image analysis software, allowing for objective assessment of marker expression and minimizing observer-related bias. Analysis of whole-slide images was performed collaboratively by four observers blinded to the patient and sampling time point using Motic SlideViewer software (Motic VM V1 Viewer 1.0) to ensure consistency in interpretation. Immunolabeled cells were evaluated in five non-overlapping high-power fields (400×) [28] selected from representative areas of the inflammatory infiltrate after examination of the entire section at low magnification. Necrotic areas were excluded from the evaluation due to their potential to produce nonspecific staining and false-positive results, as were regions with prominent vascularization, severe spongiosis, or technical artifacts related to tissue processing, sectioning, or staining. Areas with technical staining artifacts that prevented reliable discrimination of specific DAB signals from the background were excluded. Quantification was conducted using FIJI (ImageJ 2.14.0 1.54p, NIH, MD, USA). A color threshold was applied to isolate the specific chromogenic signal and distin guish it from the background. The thresholded image was then binarized to convert the selected signal into a binary format. The area occupied by positive staining was subsequently quantified and expressed as a percentage of the total image area. All images were processed using identical parameters for brightness, contrast, and thresholding to ensure consistency across samples.

### 2.5. Descriptive Analysis

The data are presented descriptively as individual values for each cat and time point. No inferential statistical analyses were performed.

## 3. Results

### 3.1. Patient Characteristics

Five client-owned mixed-breed cats with a mean age of 7 years were included. Four had the ulcerative form of FCGS, and one had the proliferative form. Cats were housed indoors or had a mixed indoor–outdoor lifestyle and lived with a mean of two additional cats per household. Individual patient characteristics and FIV/FeLV status are shown in Table A1 and Table A2, respectively.

### 3.2. Clinical Evaluation

Clinical response after PME varied among individuals; however, all cats exhibited reductions in SDAI scores by day 30 compared with baseline (Figure 2). The degree of improvement differed among cats. At D0, cats showed a mean SDAI score of 12.6. At D30, two showed good improvement, two showed moderate improvement, and one showed mild improvement. Three cats were followed up to D180, with two of them showing good improvement and one showing mild improvement at this time point. Cat E showed good improvement in SDAI score but died due to complications associated with FeLV (lymphoblastic leukemia) after the study period. Cat C was withdrawn after D90 due to kidney disease, although it showed an improvement in SDAI score after PME. Cat D was lost to follow-up after D30. Cat A received amitriptyline for feline orofacial pain syndrome (FOPS) control from D60 to D120 and then CBD oil afterwards, achieving full remission by D180. Cat C received omeprazole.

### 3.3. Blood Parameters

Three of the five cats showed anemia at baseline (Table A2, Figure S1). Persistent non-regenerative anemia was observed in cats D and E, both of which were positive for FeLV and/or FIV. In contrast, transient regenerative anemia was documented in cat C during follow-up. Monocytosis was particularly pronounced in cats D and E. Eosinophilia and increased ALP were observed in cat B at D15, which declined by D60, coinciding with the detection of endoparasites (*Dipylidium* spp.), after which the cat received anthelmintic treatment.

Hyperglobulinemia represented the most consistent biochemical abnormality and was present in all cats at one or more time points (Figure S2). Hypoalbuminemia was observed in two of five cats at D0, which subsequently normalized in all but one cat. Additionally, one cat had elevated serum urea and creatinine levels and consequently received treatment for kidney disease.

### 3.4. Histopathological Findings

Oral biopsies were collected from three FeLV-positive cats (A, D, and E) at D0 and D30 (Figure 3A). Cat B did not attend the scheduled D30 visit under conditions suitable for general anesthesia, and cat C was not biopsied because elevated serum urea and creatinine concentrations were detected. Considering the potential anesthetic risk, general anesthesia for biopsy collection was not performed in the interest of this cat’s welfare. Tissue sections exhibited epithelial hyperplasia with abundant cellular debris in the most severe cases. Moderate-to-marked lymphoplasmacytic infiltrate was consistently noted, primarily localized within the submucosa. Mott cells were occasionally observed within the infiltrate. Neutrophils were identified in the submucosa and perivascular regions, particularly in areas displaying edema and necrosis. In samples graded as most severe, there was evident spongiosis, interstitial edema, and dense inflammatory infiltrate encasing muscle fibers. Histopathologic examination of the D0 and D30 oral biopsies from cat E showed chronic inflammatory changes without evidence of neoplastic infiltration. Lymphoblastic leukemia was diagnosed after completion of the study period, on day 200.

Two cats exhibited a reduction in severity of microscopic inflammatory changes accompanied by clinical improvement (decrease in SDAI) (Figure 3B), whereas one cat showed an unchanged inflammatory score and minimal improvement in SDAI, and was positive for FIV and FeLV.

### 3.5. Immunohistochemical Findings

IHC analysis revealed distinct labeling patterns for CD20, FoxP3, and IL-17 in sequential biopsies from three cats (Figure 4a). CD20 labeling was intense and predominantly concentrated in the lamina propria, with infiltration of the submucosa. Both nuclear and cytoplasmic FoxP3 labeling was observed in a few cells within the lamina propria, mainly located near the epithelium in areas showing inflammation or edema. IL-17 labeling was detected in the cytoplasm and interstitium in the epithelium, lamina propria, and submucosa.

Quantitative analysis of immunolabeled areas demonstrated reductions in CD20 expression in all cats following PME. The magnitude of these changes varied among individuals, with decreases of 46.3%, 39.4%, and 18.6% in cats A, D, and E, respectively. Cats A and E also showed a decrease in histologic inflammatory score and clinical improvement. In contrast, FoxP3 and IL-17 immunolabeling patterns showed marked interindividual variability. FoxP3-positive areas decreased in two cats that also exhibited a reduction in both SDAI and histologic inflammatory scores, but one cat showed an increase in FoxP3 labeling area while its histologic inflammation score remained unchanged, and it showed minimal clinical improvement. IL-17 labeling decreased in two cats and increased in one cat. Individual SDAI scores, histopathologic inflammatory scores, and quantitative immunohistochemical measurements are summarized in Figure 4b.

## 4. Discussion

The present pilot prospective study provides longitudinal information regarding the clinical, histopathologic, and local immunological changes that occur following PME in cats with FCGS. Traditionally, patients require medical management for more than 14 days following dental extraction [11]. The cats showed clinical improvement 1 month post-PME, though the degree varied over time and between cats as measured by SDAI. The clinical response after dental extractions may continue to evolve over several months, and early evaluations may not reflect the final outcome. Improvement or clinical resolution has been reported six months after surgery [11,29]. Therefore, although the cats were followed for up to 180 days, this study primarily aimed to characterize early post-PME changes, particularly during the first 30 days. Previous reports using different scoring systems observed that 52% of cats showed clinical improvement 38 days after surgical treatment [29], while more recent studies have reported significant improvement at 60 days [18].

In this small cohort, the cats positive for retroviral infections exhibited the most pronounced hematologic abnormalities. The main hematologic finding was anemia, observed predominantly in cats positive for FIV and/or FeLV. These findings are consistent with recent reports of anemia in 18% of FeLV and 12% of FIV-positive cats, as well as leukemia in 16% and 3% of these populations, respectively [30]. The same study reported FCGS in 11% of FeLV-positive cats and 24% of FIV-positive cats. Among the three FeLV-positive cats, cats D and E developed clinically relevant hematologic complications during follow-up. Despite FeLV positivity, cats A and E showed clinical improvement (improved SDAI score). FeLV infection has been associated with a 7.5-fold higher likelihood of poor clinical response after dental extraction [13]. In this study, eosinophilia and increased ALP were observed in one patient at D15 post-PME, possibly associated with its concurrent *Dipylidium* spp. infection. Although eosinophilia is not commonly reported in cats with FCGS, a retrospective study documented peripheral eosinophilia in approximately 25% of cases [31], highlighting the importance of investigating concurrent infections.

The main biochemical finding at D0 was hyperglobulinemia, consistent with previous reports describing chronic antigenic stimulation and systemic inflammatory activation in cats with FCGS [5,32]. However, no changes were observed post-PME. Recent studies suggested that dental extraction does not contribute to a significant decrease in alpha-1 acid glycoprotein levels at D60, indicating the maintenance of systemic inflammation despite clinical improvement [18]. In contrast, our results showed a correction of serum albumin levels 15 days after PME in all but one cat. The post-surgical increase in serum albumin levels may reflect a reduction in systemic inflammation following PME, as albumin is a prominent negative acute-phase protein in cats, and decreased concentrations are observed in chronic inflammatory diseases [33]. These contrasting results emphasize the need for further investigation into the effects of PME on the cat immune system.

In this study, one cat showed increased urea and creatinine levels compatible with chronic kidney disease (CKD). Previous reports found that approximately 10% of cats with FCGS presented with CKD [34]. Later reviews highlighted the importance of screening for concurrent systemic disorders, as comorbidities such as CKD may influence both clinical severity and treatment response [4,5]. A recent cross-sectional analysis comparing cats with caudal stomatitis and healthy controls revealed mild but significant differences in biochemical markers, such as lower creatinine and higher phosphorus levels, without a higher prevalence of CKD [35]. However, previous studies found that 26.5% of cats showed serum urea concentrations above the upper reference limit [31]. These conflicting results indicate that we should consider monitoring renal function biomarkers in cats with FCGS.

The histologic findings showed the classical lymphoplasmacytic and neutrophilic infiltrate mentioned in previous reports [5,9,32,36]. Area-based quantification was used to provide a standardized assessment of the overall extent of immunolabeling within the inflammatory infiltrate, particularly in regions with dense or partially confluent staining where reliable identification of individual cell boundaries was not always possible. The same approach was applied consistently across all markers. The results showed a reduction in the histologic inflammatory infiltrate score in two cats and a reduction in CD20 immunolabeling in three cats. However, CD20 immunolabeling decreased to different degrees in the three cats, irrespective of their SDAI response, which raises the question of whether it reflects the removal of the extraction-related antigenic stimulus rather than control of the disease. Interestingly, the changes in FoxP3 and IL-17 immunolabeling were heterogeneous: two cats exhibiting histologic improvement showed reductions in FoxP3-positive areas, while the cat with persistent severe histologic inflammation demonstrated increased FoxP3 immunolabeling despite minimal clinical improvement. Although the biological significance of this observation remains uncertain and any interpretation is necessarily speculative, decreased FoxP3 immunolabeling could be consistent with reduced antigenic stimulation following PME, whereas increased FoxP3 immunolabeling could reflect a compensatory response to persistent inflammation. Furthermore, since IHC detection provides expression and morphologic information, it does not allow for the assessment of cellular functionality. IL17-A showed marked interindividual variability. Previous transcriptomic studies on FCGS suggest that signaling pathways related to the IL-17 family may be involved in the local immune response [2,8,10]. However, these findings are not directly comparable with the immunohistochemical observations of the present study. Elevated pro-inflammatory cytokines, such as IL-6, TNF-α, and IFN-γ, have been documented in FCGS and constitute part of an inflammatory milieu that promotes the differentiation and function of Th17 cells [10,32]. The detection of these cytokines using immunolabeling techniques has not been performed in caudal oral mucosa. Although IHC detection of IL-17A has previously been reported in other feline tissues using a different antibody [27], this does not constitute validation of the antibody used in the present study for feline tissue. Therefore, the IL-17A IHC findings should be interpreted with caution.

The main limitations of this pilot study include the small sample size and the availability of paired histopathologic and IHC samples from only three cats. Because paired sections were not always processed within the same IHC run, between-run variability in chromogen development and staining intensity cannot be excluded. Sequential biopsies were obtained from contralateral sides at D0 and D30 to avoid healing tissue; therefore, site-related differences in tissue composition may have influenced the area-based IHC measurements. Interobserver agreement was not formally assessed, although final histopathologic scores were assigned by consensus among the four blinded observers. Furthermore, it should be noted that, by chance, the three cats biopsied were FeLV-positive, which may negatively affect the lymphocyte populations and the clinical response following dental extraction [13]. Another limitation of this pilot study is that feline calicivirus (FCV) was not tested because a validated FCV assay was not available at our institution. Meloxicam was administered during the early postoperative period and discontinued before the D30 biopsy; nevertheless, a potential influence on inflammatory or immune-related markers at D30 cannot be completely excluded. Because this study involved client-owned cats, concomitant medical treatment could not be withheld when clinically indicated, particularly for pain management and animal welfare. In addition, one cat developed FOPS during follow-up and subsequently received additional medication that could have potentially influenced the immune-related outcomes in a manner that was not controlled for by the study design. However, these additional treatments were initiated after the D30 biopsy and therefore should not affect the early histopathologic and IHC comparisons between D0 and D30. Nevertheless, their potential influence should be considered when interpreting the later clinical and immunologic findings.

A limited number of studies have reported clinical follow-up after PME in cats with FCGS. Notably, studies on cell therapy have provided valuable insight into the immunological evolution of cats with refractory FCGS treated with MSC [20,21,22]. However, these findings should not be directly extrapolated to cats undergoing PME because MSC therapy represents an active immunomodulatory intervention, whereas PME primarily reduces local antigenic stimulation. This lack of comparable data also highlights the relevance of investigating immunologic changes following the recommended standard-of-care surgical treatment. To the best of our knowledge, the present pilot study is among the few that prospectively evaluated the longitudinal clinical, histopathological, and local immunological changes that occur after PME. A better understanding of the immune changes induced by PME could provide critical insights into the pathophysiology of the disease and help explain the heterogeneity in clinical outcomes. Larger longitudinal studies are needed to determine whether specific immunological patterns are associated with treatment outcomes.

## 5. Conclusions

Clinical SDAI scores decreased after PME in the five cats, although the magnitude and duration of improvement varied among individuals. In the three FeLV-positive cats with paired biopsies, CD20 immunolabeling decreased irrespective of the clinical and histologic responses, whereas FoxP3 and IL-17 levels showed variable changes. These findings represent preliminary descriptive observations of early tissue changes following PME and should be interpreted considering the small biopsy subset and the methodological limitations of this study.

## Figures and Tables

**Figure 1:**
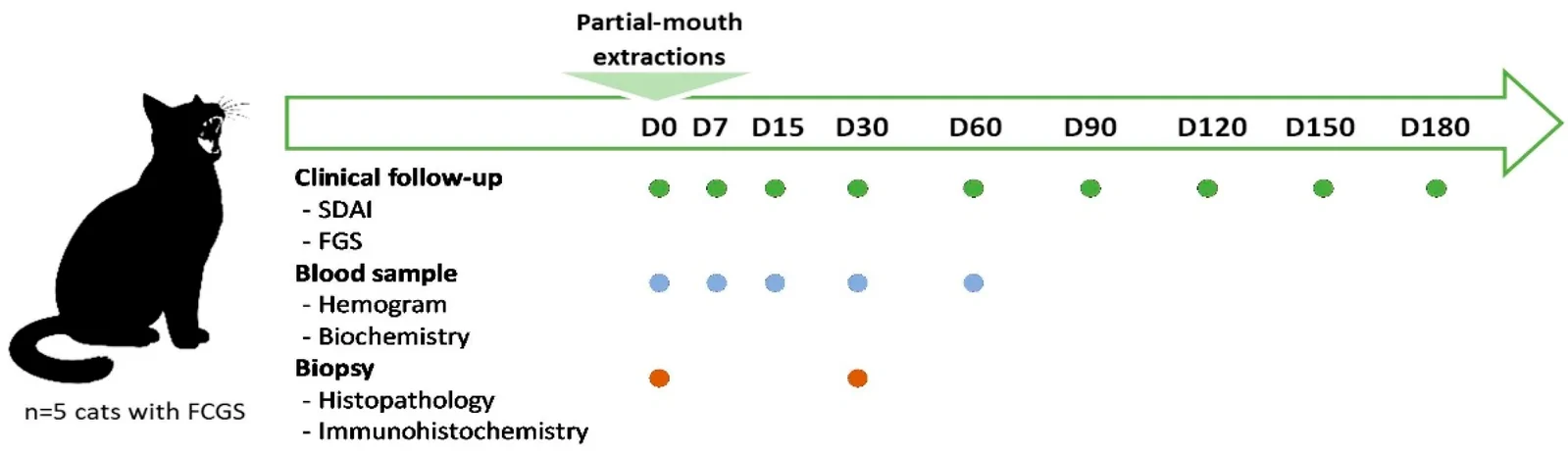
Overview of the study design.

**Figure 2:**
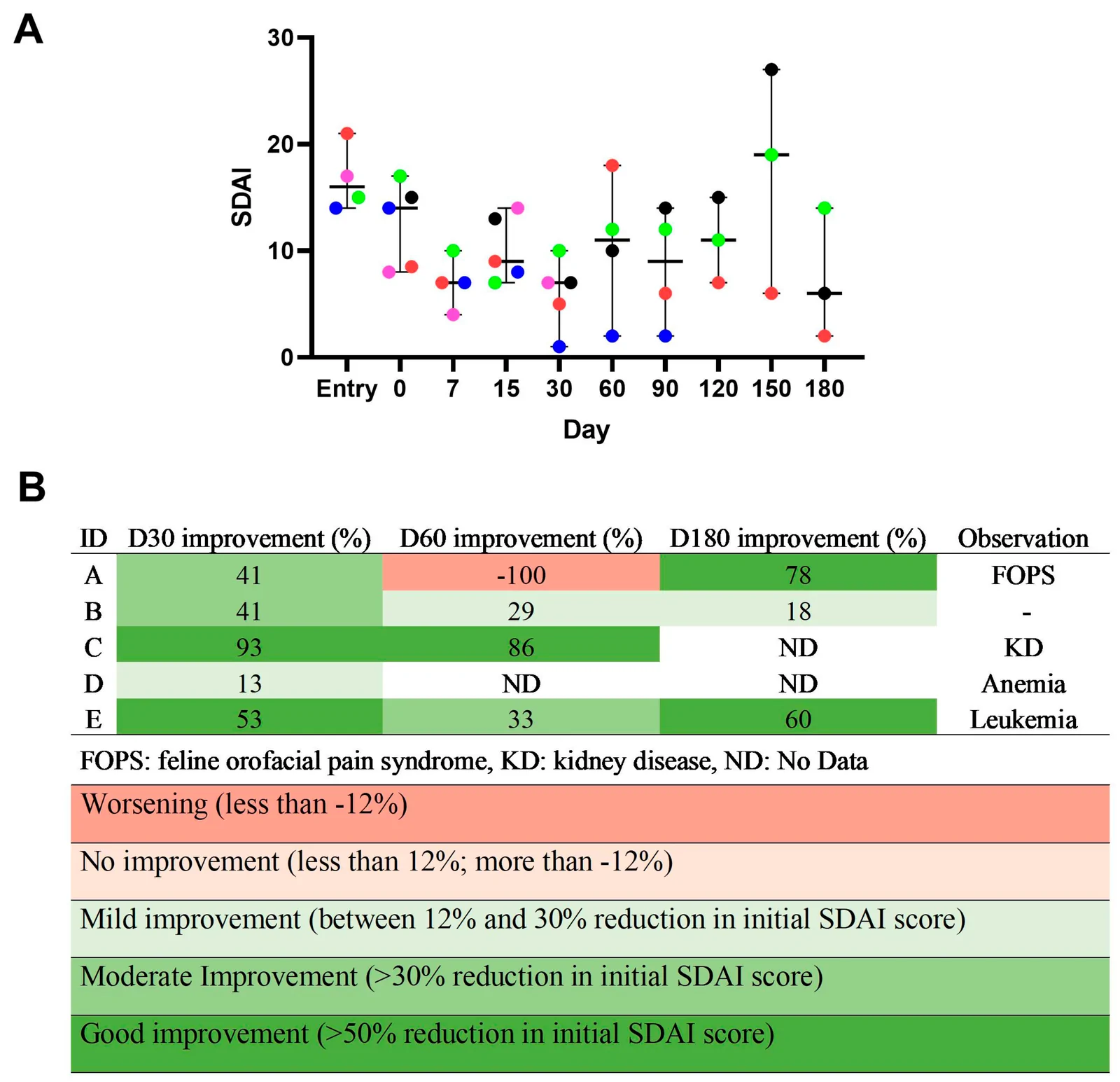
(**A**) Stomatitis Disease Activity Index (SDAI) for each cat and timepoint. Each colored dot represents a single feline patient, coded as follows: A (red), B (green), C (blue), D (pink), and E (black). The horizontal black line indicates the median, and the whiskers indicate the minimum and maximum values. ND: no data. (**B**) Percentage change in SDAI over time and additional observations for each cat. The color scale represents clinical changes, with green indicating improvement and red indicating worsening.

**Figure 3:**
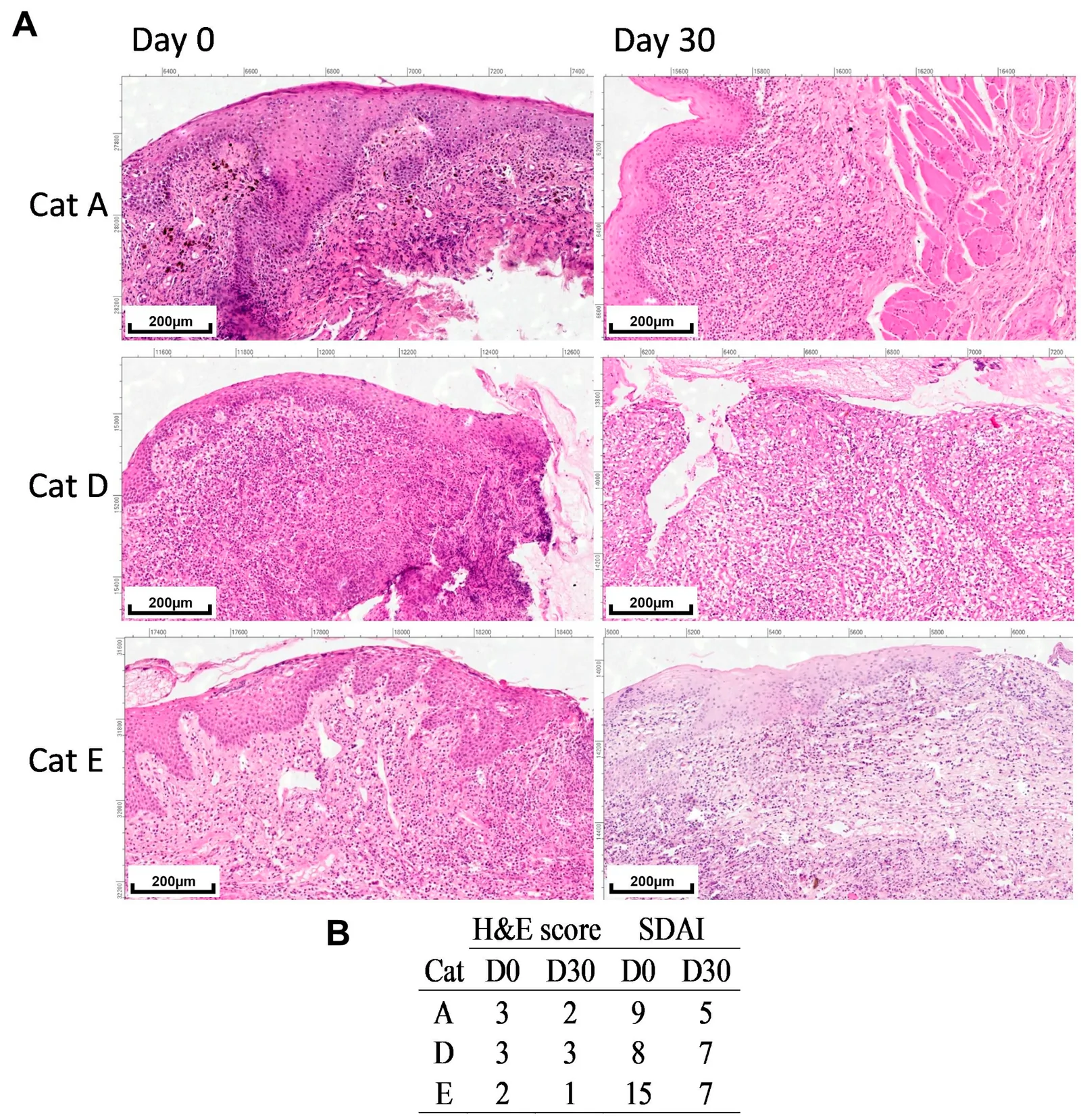
Inflammatory changes in oral biopsies pre- and post-PME. (**A**) Microscopic images of oral biopsies (H&E, 100×, scale bar = 200 μm). (**B**) Score for severity of microscopic inflammatory changes and SDAI scores.

**Figure 4:**
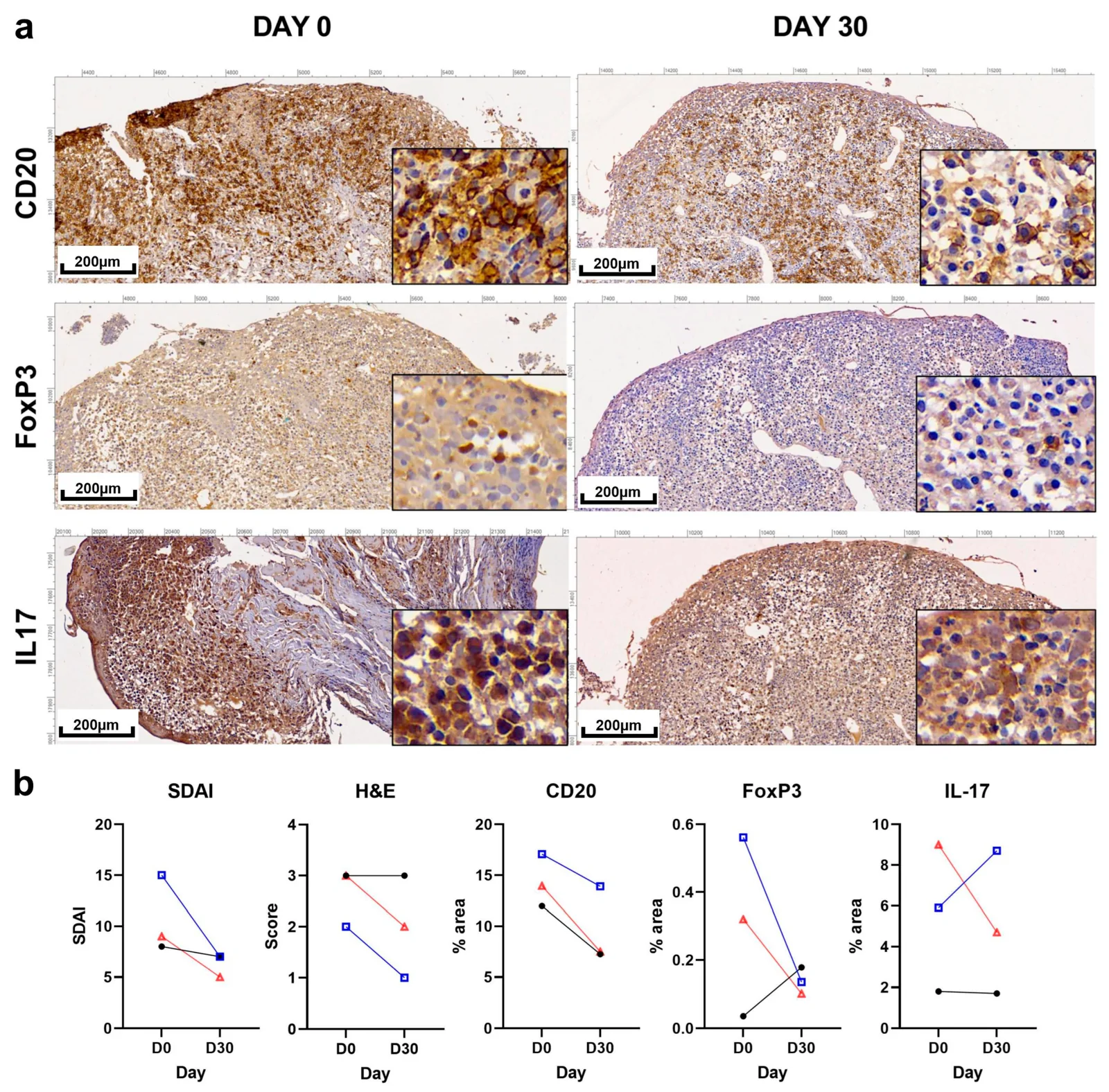
Immunohistochemistry images. (**a**) Illustrative microscopic images (100×) of immunohistochemical staining for CD20, FoxP3, and IL-17 in oral mucosa biopsies at days 0 and 30 from cat A. Insets (400×) show higher-magnification details of immunolabeling. (**b**) Individual SDAI scores, severity of microscopic inflammatory scores (H&E), and mean percentage of immunolabeled area for each marker. Each symbol/line represents a single feline patient, coded as follows: A (red), D (black), and E (blue).

**Table 1:** Details of antibodies for immunohistochemical labeling.

Primary Antibody	Manufacturer	Dilution
Rabbit anti-CD20 (polyclonal)	Invitrogen/Catalog # PA5-16701	1:400
Mouse anti-FoxP3 (clone 221D)	Donated by Giovanna Roncador, Spanish National Cancer Research Center (CNIO)	undiluted
Rabbit anti-IL-17A (polyclonal)	ABCAM/Catalog # ab79056	1:200

## Data Availability

The original contributions presented in this study are included in the article/Supplementary Material. Further inquiries can be directed to the corresponding author.

## Supplementary Materials

The following supporting information can be downloaded at: https://www.mdpi.com/article/10.3390/vetsci13101038/s1, Figure S1. This figure displays individual data points for key hematological parameters: red blood cells, hemoglobin, hematocrit, leukocytes, lymphocytes, neutrophils, monocytes, and eosinophils. Measurements were conducted at specified time points from day 0 to day 60. Each individual colored dot represents a single feline patient, coded as follows: A (Red), B (Green), C (Blue), D (Pink), and E (Black). Error bars indicate the standard deviation (SD) of the mean, and the dashed horizontal lines denote the established reference range for each respective parameter. Figure S2. This figure displays individual data points for key biochemistry parameters: serum globulin, albumin, total proteins, alkaline phosphatase (ALP), urea and creatinine. Measurements were conducted at specified time points from Day 0 to Day 60. Each individual colored dot represents a single feline patient, coded as follows: A (Red), B (Green), C (Blue), D (Pink), and E (Black). Error bars indicate the standard deviation (SD) of the mean, and the dashed horizontal lines denote the established reference range for each respective parameter.

## Appendix A

**Table A1:** Characteristics of cats with feline chronic gingivostomatitis.

Id	Age	Sex	Cohabiting Cats	FCGS Type	Life Style
A	8	Male	1	Ulcerative	Mixed
B	4	Female	1	Proliferative	Indoor
C	9	Male	6	Ulcerative	Indoor
D	7	Male	4	Ulcerative	Mixed
E	7	Male	0	Ulcerative	Mixed

**Table A2:** Anemia status over time for each feline patient.

ID	D0	D7	D15	D30	D60	D90	D180	Anemia Type	FIV	FeLV
A	NO	NO	NO	NO	-	-	-	-	NEG	POS
B	NO	NO	NO	NO	+	-	NO	-	NEG	NEG
C	++	+	NO	NO	-	NO	-	R	POS	NEG
D	+++	+	+	++	-	-	-	NR	POS	POS
E	+	-	+	++	NO	+	+++	NR	NEG	POS

Anemia status is indicated as NO (Absence of Anemia) or classified by severity: Mild (+), Moderate (++), or Severe (+++). The Anemia Type column classifies the bone marrow response as Regenerative anemia (R) or Non-Regenerative anemia (NR). Serological results for the Feline Immunodeficiency Virus (FIV) and feline leukemia virus (FeLV) are shown as Positive result (POS) or Negative result (NEG). A hyphen (-) indicates that the data was not collected or is not available.
