# Supplementary material for: Longitudinal Clinical, Histopathologic, and Immunologic Findings in Cats with Feline Chronic Gingivostomatitis Following Partial-Mouth Extractions: A Pilot Prospective Study
Source: Vet Sci. 2026 Oct 1;13(10):1038. doi: 10.3390/vetsci13101038 (PMC13709930; doi:10.3390/vetsci13101038)
Supplement: Supplementary file 1 [file vetsci-13-01038-s001.zip › vetsci-4518025-supplementary.pdf]

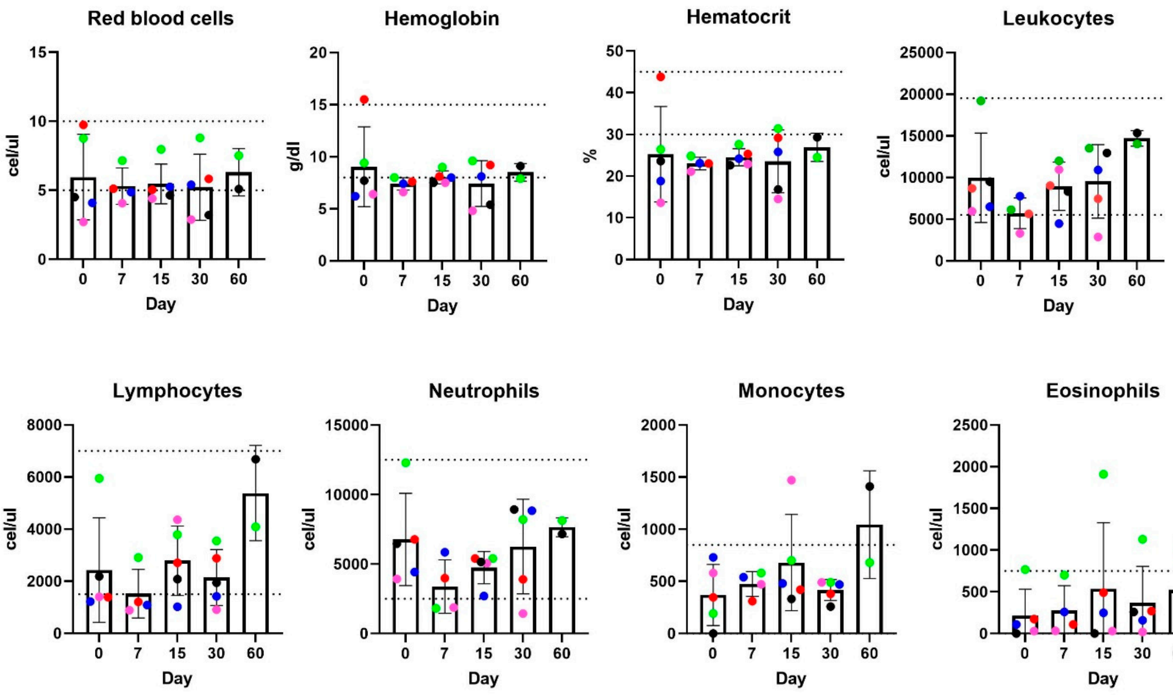

Figure S1. This figure displays individual data points for key hematological parameters: red blood cells, hemoglobin, hematocrit, leukocytes, lymphocytes, neutrophils, monocytes, and eosinophils. Measurements were conducted at specified time points from day 0 to day 60. Each individual colored dot represents a single feline patient, coded as follows: A (Red), B (Green), C (Blue), D (Pink), and E (Black). Error bars indicate the standard deviation (SD) of the mean, and the dashed horizontal lines denote the established reference range for each respective parameter.

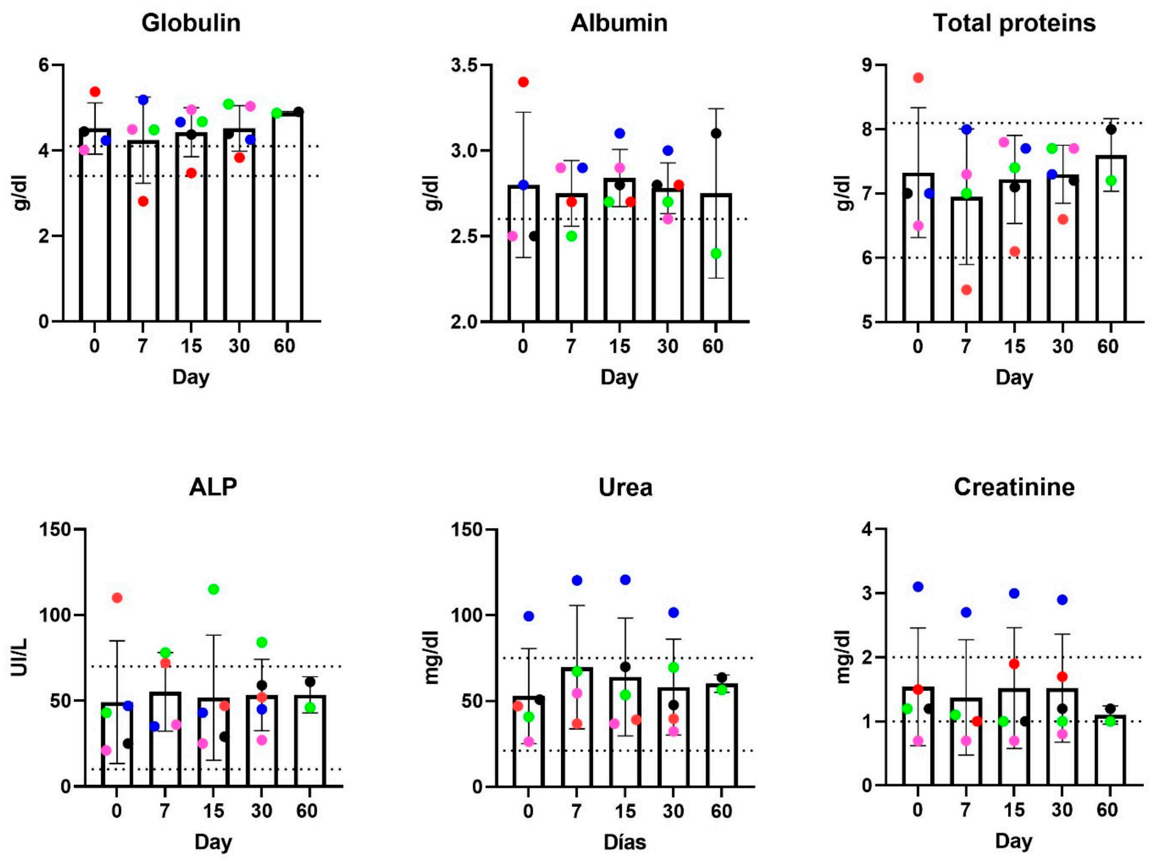

Figure S2. This figure displays individual data points for key biochemistry parameters: serum globulin, albumin, total proteins, alkaline phosphatase (ALP), urea and creatinine. Measurements were conducted at specified time points from Day 0 to Day 60. Each individual colored dot represents a single feline patient, coded as follows: A (Red), B (Green), C (Blue), D (Pink), and E (Black). Error bars indicate the standard deviation (SD) of the mean, and the dashed horizontal lines denote the established reference range for each respective parameter.
